# Fractional integral-like processing in retinal cones reduces noise and improves adaptation

**DOI:** 10.1371/journal.pone.0205099

**Published:** 2018-10-04

**Authors:** Antal Martinecz, Mihoko Niitsuma

**Affiliations:** Department of Precision Mechanics, Chuo University, Tokyo, Japan; China University of Mining and Technology, CHINA

## Abstract

In the human retina, rod and cone cells detect incoming light with a molecule called rhodopsin. After rhodopsin molecules are activated (by photon impact), these molecules activate the rest of the signalling process for a brief period of time until they are deactivated by a multistage process. First, active rhodopsin is phosphorylated multiple times. Following this, they are further inhibited by the binding of molecules called arrestins. Finally, they decay into opsins. The time required for each of these stages becomes progressively longer, and each stage further reduces the activity of rhodopsin. However, while this deactivation process itself is well researched, the roles of the above stages in signal (and image) processing are poorly understood. In this paper, we will show that the activity of rhodopsin molecules during the deactivation process can be described as the fractional integration of an incoming signal. Furthermore, we show how this affects an image; specifically, the effect of fractional integration in video and signal processing and how it reduces noise and the improves adaptability under different lighting conditions. Our experimental results provide a better understanding of vertebrate and human vision, and why the rods and cones of the retina differ from the light detectors in cameras.

## Introduction

As humans rely heavily on visual perception, research on human vision is currently receiving particularly strong interest. Thus, the rods and cones of the retina became among the most well researched cells in human physiology. However, despite the rich literature and constant progress in this field, human vision is still not understood in its entirety owing to its overall complexity [1–5]. This fact is well illustrated by the different scales of interactions that are required to produce a signal in the retina: (i) molecular processes within the photoreceptor cells [6–8]; (ii) the various roles of the photoreceptor cells [9, 10]; (iii) their interactions with other cells before the signal leaves the retina [11–13].

In this paper, we focus on the first step of the signal forming process of rods and cones: the activation and deactivation of rhodopsin. These proteins enter their active state upon impact with a photon, which in turn activates the rest of the signalling cascade until they are deactivated by a multistage process. Each stage of the deactivation process greatly reduces their activity; however, the time required to complete each step progressively increases, resulting in the temporary accumulation of partially deactivated rhodopsin molecules that still show some residual activity. It is currently unclear whether (and how) these residual activities affect the signal produced by the cell.

During a previous conference, we reported (as preliminary results) that the structure of this process has the potential to approximate the mathematical operations of fractional integration. [14]. Furthermore, we have also shown that the phosphorylation process can approximate this kind of behaviour, based on the commonly used models of the cones [15–17].

Fractional integrals generalise traditional Riemann integrals by allowing integration of non-integer times (e.g. half-integrals). Fractional calculus, which also encompasses fractional integrals, has many interesting real-world applications in various fields, such as robotics [18], modelling ground water pollution [19], modelling drug diffusion in the human body [20], modelling the dynamics of neurons [21], and modelling protein dynamics [22]. Moreover, fractional calculus has been gaining traction in, and proved to be a useful tool for, our topic of interest: image and signal processing [23–25].

In this paper, we investigate whether the multi-stage deactivation process of rhodopsin and related residual activities offer any signal processing benefits, and how it affects signals in general. In our previous work, we used the model presented in [15] to show that this process has the potential to approximate fractional integral-like behaviour. To investigate the effect of the deactivation process, we have expanded this model with the activity of arrestin bound rhodopsin, as it was not previously included.

In addition, we show that the activity of rhodopsin still approximates fractional integration after the addition of the arrestin binding process to the cone model. Furthermore, the addition of the arrestin binding process model expands the frequency range of the approximation. Our main purpose for including these results is to demonstrate that residual activities can accumulate in signalling processes; therefore, they should not be neglected. Finally, as the activity itself can be described as fractional integration, its effects can be predicted without explicitly modelling the process.

## Materials and methods

### Mathematical model

Active rhodopsin is constantly deactivated by the following process: first, rhodopsin is phosphorylated 5-7 times in rapid succession; following this, it is inhibited by arrestin before finally decaying into opsins within the next few seconds [26–29]. Phosphorylation rates exponentially decrease with each successive phosphorylation: *γ*_*i*_ ≈ *γ* ⋅ 0.9^*i*^, where *γ*, is the rate of the first phosphorylation [15]. However, the rate of arrestin binding linearly increases with each phosphorylation: *β*_*i*_ ≈ *i* ⋅ 0.5, where *β*_*i*_, is the rate of arrestin binding to rhodopsin phosphorylated *i* times. This process is described and modelled in detail in [15–17], which we used as a foundation for our model (Eqs 1a–1d). We extended the model by adding the stage where rhodopsin is inhibited by arrestin, and retains only a fraction of its original activity (Eq 1e) [10, 30–33]. With each phosphorylation, rhodopsin is inhibited by 50% [15], and the binding of arrestin further inhibits activity by *a* = 50–90% [30–32].

The equations of the model for 6 phosphorylations are as follows:

$$\begin{array}{ccc} {\dot{r}}_{0} & = & \text{input}\left(t\right)-\gamma_{0}r_{0} \end{array}$$ (1a)

$$\begin{array}{ccc} {\dot{r}}_{1} & = & \gamma_{0}r_{0}-\left(\gamma_{1}+\beta_{1}\right)r_{1} \end{array}$$ (1b)

$$\begin{array}{c} \begin{array}{cc} {\dot{r}}_{2} & =\gamma_{1}r_{1}-\left(\gamma_{2}+\beta_{2}\right)r_{2} \end{array}\\ \vdots \end{array}$$ (1c)

$$\begin{array}{ccc} {\dot{r}}_{6} & = & \gamma_{5}r_{5}-\beta_{6}r_{6} \end{array}$$ (1d)

$$\begin{array}{ccc} {\dot{r}}_{arr} & = & \sum_{i=0}^{6}\beta_{i}r_{i}-0.3r_{arr}, \end{array}$$ (1e)

where *r*_*i*_ is the number of rhodopsin molecules with levels of phosphorylation and *r*_*arr*_ is the number of rhodopsin molecules bound by arrestin. The total activity of the rhodopsin is the output of the model, as follows:

$$\begin{array}{ccc} \text{output}(t) & = & \sum_{i=0}^{6}2^{-i}r_{i}+2^{-6}\cdot a\cdot r_{arr} \end{array}$$ (2)

**Fig 1:**
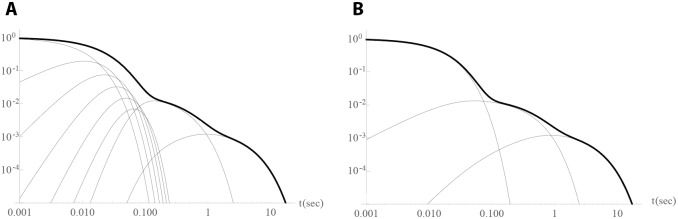
Impulse responses of full (panel A, Eqs 1 and 2) and simplified model (panel B, Eqs 3 and 4). The thin lines are the contributions of the individual equations to the output (black curves).

The arrestin binding rates for the intermediate phosphorylation steps are of little significance, as the phosphorylation rates are a magnitude faster. Therefore, as previously reported in [14], the system of equations can be simplified without affecting the output. Fig 1 shows that the impulse responses were approximately the same before and after simplification. As these are linear systems of equations, estimating the impulse response was sufficient for approximation.

The simplified equations are as follows:

$$\begin{array}{ccc} {\dot{r}}_{0} & = & \text{input}\left(t\right)-\gamma_{5}r_{0} \end{array}$$ (3a)

$$\begin{array}{ccc} {\dot{r}}_{6} & = & \gamma_{5}r_{0}-\beta_{6}r_{6} \end{array}$$ (3b)

$$\begin{array}{ccc} {\dot{r}}_{arr} & = & \beta_{6}r_{6}-0.3r_{arr}, \end{array}$$ (3c)

In this case the output is:

$$\begin{array}{ccc} \text{output}(t) & = & r_{0}+2^{-6}r_{6}+2^{-6}a\cdot r_{arr} \end{array}$$ (4)

## Fractional integrals

### Definition

Fractional integrals require multiple definitions, approximations, and numerical methods to solve [34–39]. Here, we used the Riemann-Liouville definition expressed with a convolution operation [40] as the definition, which shows the impulse response of the operation. In this paper, we show how this impulse response was approximated by the deactivation of rhodopsin in response to a single impulse of light.

$$\begin{array}{c} I^{\alpha }f\left(t\right)=f\left(t\right)⊗\left(\frac{1}{\Gamma (\alpha )}t^{\alpha -1}\right). \end{array}$$ (5)

The above equation is a linear operation with an impulse response of $\frac{1}{\Gamma (\alpha )}t^{\alpha -1}$, where Γ(*α*) is the gamma function and the generalisation of the factorials.

### Approximation

**Fig 2:**
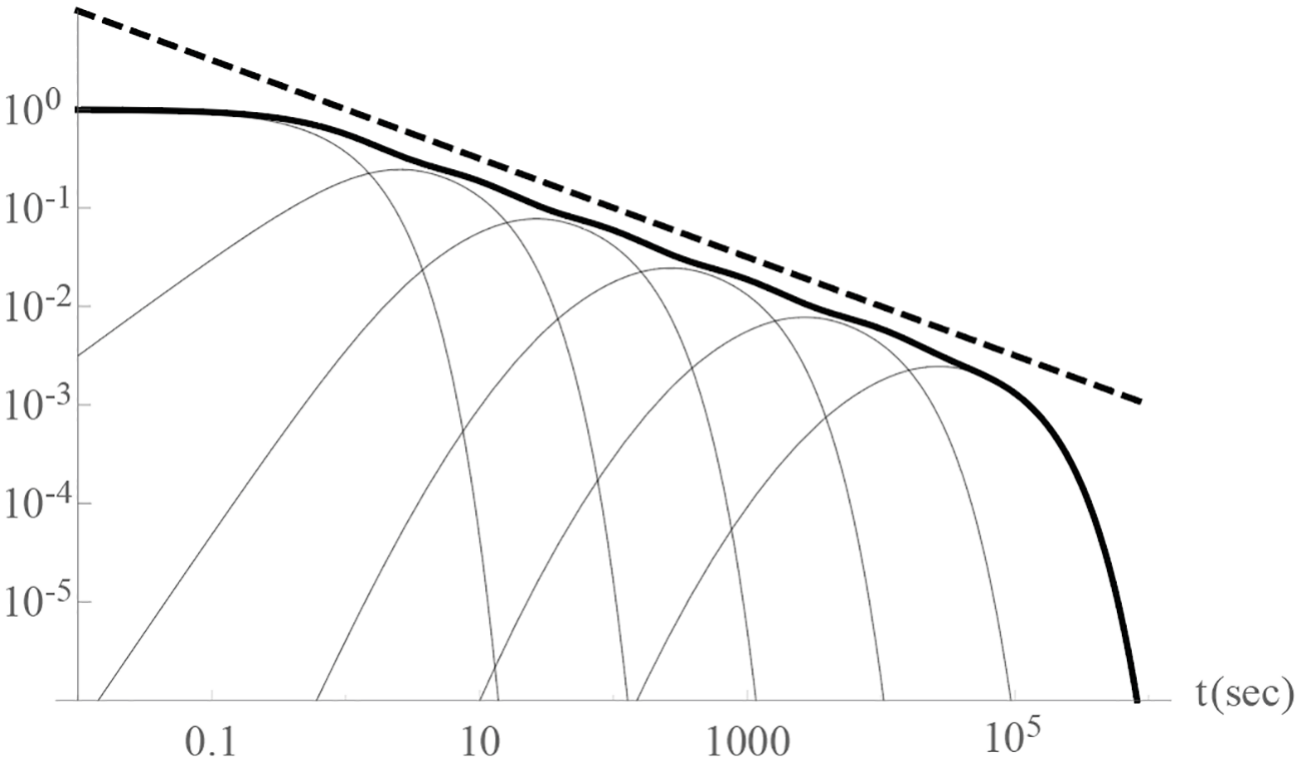
Approximation (solid black curve, Eqs 6–9) of a half integral’s (I^0.5^) impulse response: *t*^−0.5^ (dashed line) on the log–log plot. The grey lines represent each feedback loop’s contribution to the output.

As mentioned above, linear operations and systems are fully defined by their impulse responses. A system whose impulse response approximates the impulse response of a fractional integral will also approximate the fractional integral itself. Similar to results reported in [14] and [21], we have shown that connected feedback loops with logarithmically decreasing poles (Eqs 6–9) can be used to approximate fractional integrals, and the sum of their weighted responses approximates the response of the fractional integrals. (see Fig 2).

With differential equations:

$$\begin{array}{ccc} {\dot{x}}_{0} & = & f\left(t\right)-cx_{0} \end{array}$$ (6)

$$\begin{array}{ccc} {\dot{x}}_{1} & = & cx_{0}-c^{2}x_{1} \end{array}$$ (7)

$$\begin{array}{c} \begin{array}{ccc} {\dot{x}}_{2} & = & c^{2}x_{1}-c^{3}x_{2}, \end{array}\\ \vdots \end{array}$$ (8)

where the output is:

$$\begin{array}{c} I^{\alpha }f\left(t\right)=\sum_{i}\left(c^{i(1-\alpha )}x_{i}\right), \end{array}$$ (9)

and 0 < *c* < 1 is the spacing of the feedback loops; for example, *c* = 1/10.

### Bode Plots

Bode plots fully define a system by plotting the relationship between the input’s frequency and the output’s phase and amplification in linear systems. These plots can be used to identify fractional integrals, as a fractional integral I^*α*^ has a constant phase shift at −90*α* degrees and an amplification of −20*α* dB/dec [41].

**Fig 3:**
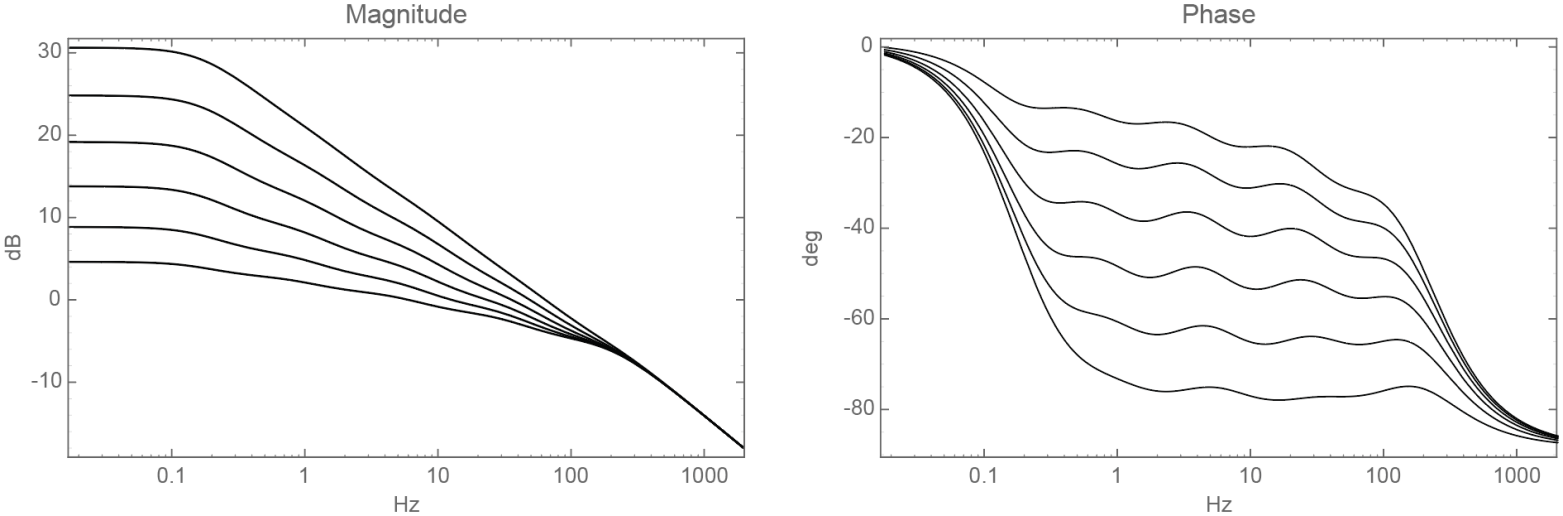
Bode plots of approximations to fractional integrals: *I*^0.15^, *I*^0.3^, *I*^0.45^, *I*^0.6^, *I*^0.75^, *I*^0.9^ (Eqs 6–9).

See examples on the approximation (Eqs 6–9) in Fig 3.

## Results and discussion

### Activity of rhodopsins approximate a fractional integral

**Fig 4:**
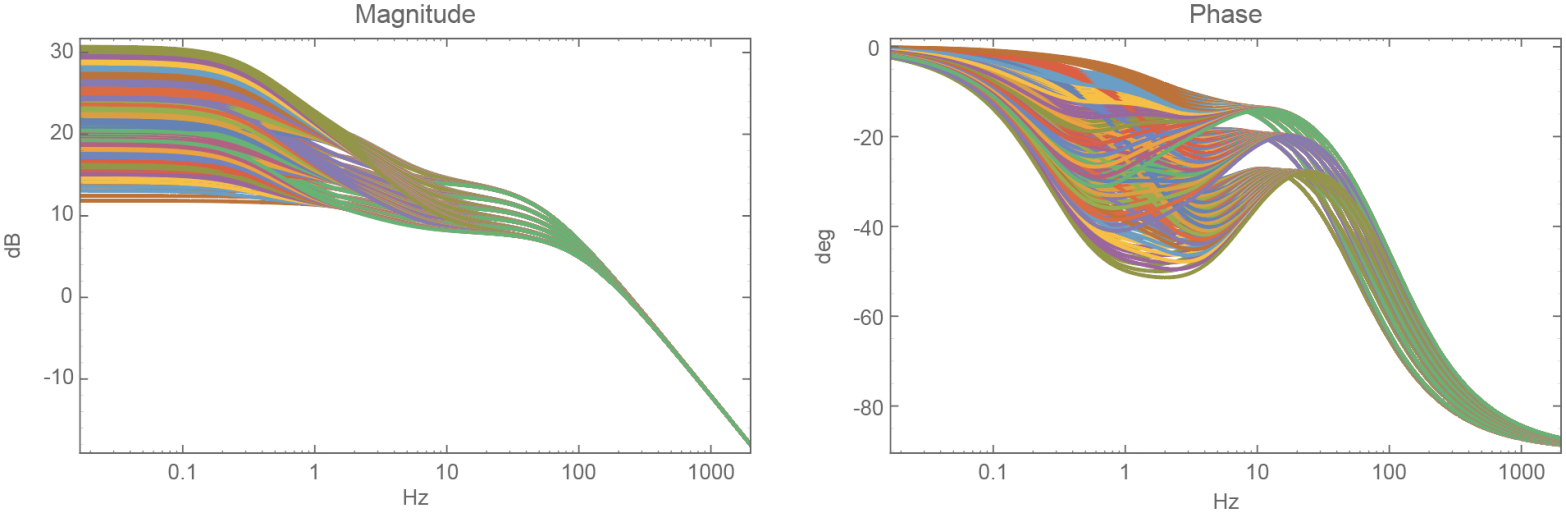
Bode-plots of rhodopsin’s activity with the different combinations of parameters found in the literature (number of phosphorylations, phosphorylation rate, inhibition by arrestin). In most of these cases, the phase-shift plots plateaued between the frequencies of 0.3-30Hz; therefore, we approximated fractional integrals within that range.

We plotted all the Bode plots (Fig 4) of the model (Eqs 3 and 4) with all the different combinations along with the published parameter ranges of the activation and deactivation rates (Table 1). As with Fig 3, the phase plots plateaued at −90*α* degrees; specifically, between −9 and −27 degrees. Therefore, the rhodopsin’s activity approximated a fractional integration between the orders of 0.1–0.3.

**Table 1:** Parameters for the rhodopsin’s deactivation process.

Parameter	Approximate values	References
Phosphorylation rates	60–90[1/*s*]	[15, 28]
Inhibition per phosphorylation	50%	[15, 28]
Number of phosphorylations	5–7	[15–17, 26]
Arrestin binding rates	2[1/*s*]	[15, 28]
Inhibition by arrestin	50%–99%	[30–32]
Decay rate into opsins	0.3[1/*s*]	[10, 33]

The parameters used for plotting are:

- phosphorylation rates between 50 and 100 1/s with steps of 10 1/s,
- inhibition by the binding of arrestin, between 51% and 99% with the steps of 5%,
- number of phosphorylations: 5, 6 or 7.

### Noise reduction and movement

**Fig 5:**
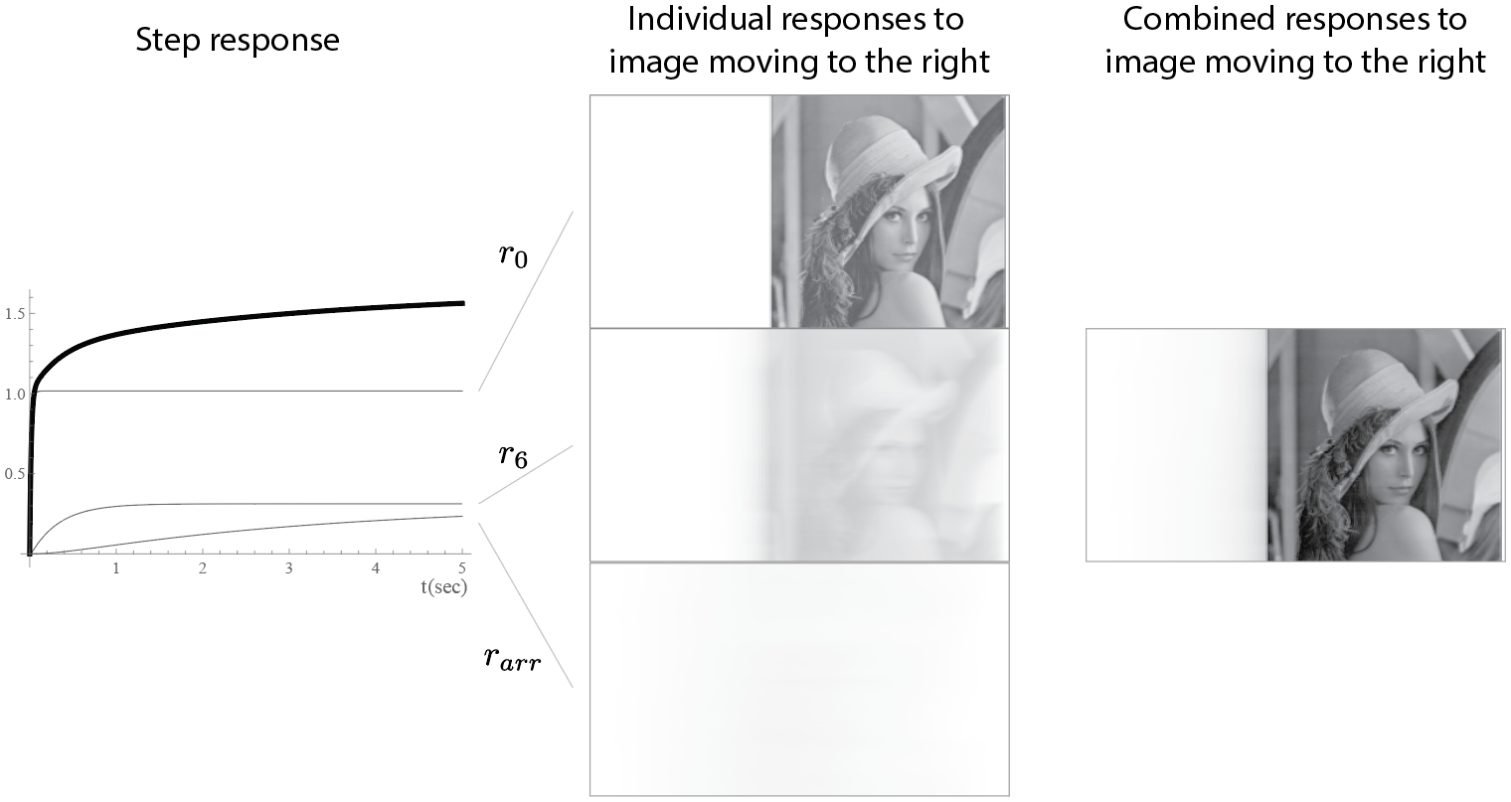
The contribution of the different stages of rhodopsin deactivation to the impulse response. The left side depicts the responses (grey) and their sum (black) to a step input. The middle images show the responses to a video of a moving image at each stage (from left to right). The right side shows the combined images.

The image produced by rhodopsin molecules can be imagined as a composition of images produced by cameras with different exposure times. Speaking mathematically, simple cameras with different exposure times can be described as a feedback loop where the exposure time corresponds to the time constant of the loop; in our case, the time rhodopsin spends at each deactivation step (see Fig 5).

In signal and image processing, feedback loops are low-pass filters; they suppress high frequency components of the signals. Thus, they are often used to reduce measurement noise. Higher frequency components in video processing are either fast movements or noise; therefore, noise reduction comes with the compromise of losing detail in moving objects. In other words, the faster the movement (higher frequency), the more detail is suppressed and blurred. In noise reduction, fractional integration offers a compromise, as it is a combination of different feedback loops and the high frequency components are kept but are slightly suppressed by the other loops. As a result, the noise reduction and blur effects are more “gentle” than in the case of feedback loops (see Fig 5). This is indicated in the bode-plots with the slope of −20*α* dB/dec. For cones this is approximately −4 dB/dec); for the first order feedback loops this is −20 dB/dec slope (Fig 4).

We speculate that, as human eyes fixate on objects of interest [42], this kind of processing allows us to visually ignore some of the motion and noise we are not interested in at a given moment. For example, during a snowfall, individual snowflakes do not necessarily disappear from our vision, but are gently suppressed.

### Adaptation

In [43], it was shown that adding a power law dynamic to an auditory-nerve and inner hair cell model allowed the adaptive part of the model to adapt to a wider range of signals. Moreover, it showed a possible explanation as to how the neurons in the auditory nerve system can adapt their responses according to input history.

In our case, fractional integrals add power law dynamics to the model, as their impulse response follows power law dynamics. Thus, the response to sustained inputs can reach higher levels than would be possible with only an exponential decay (see Fig 5). In addition, as pupils contract in response to light, the possible magnitudes are restricted (under normal lighting conditions). Therefore, power law dynamics provided by fractional integrals can allow the process to reach higher levels of overall activity in response to sustained inputs that would otherwise be impossible to achieve. This would allow the processes inside and outside cones to adapt to (and differentiate between) lighting conditions and temporarily high input levels.

### Conclusions and future directions

We have shown how rhodopsin’s ability to activate the rest of the signalling approximates a fractional integral. Furthermore, we have shown how this affects the rest of the signalling process; namely, it improves the cone cells’ abilities to adapt to different light conditions and reduces noise in “measuring” the number of incoming photons.

**Fig 6:**
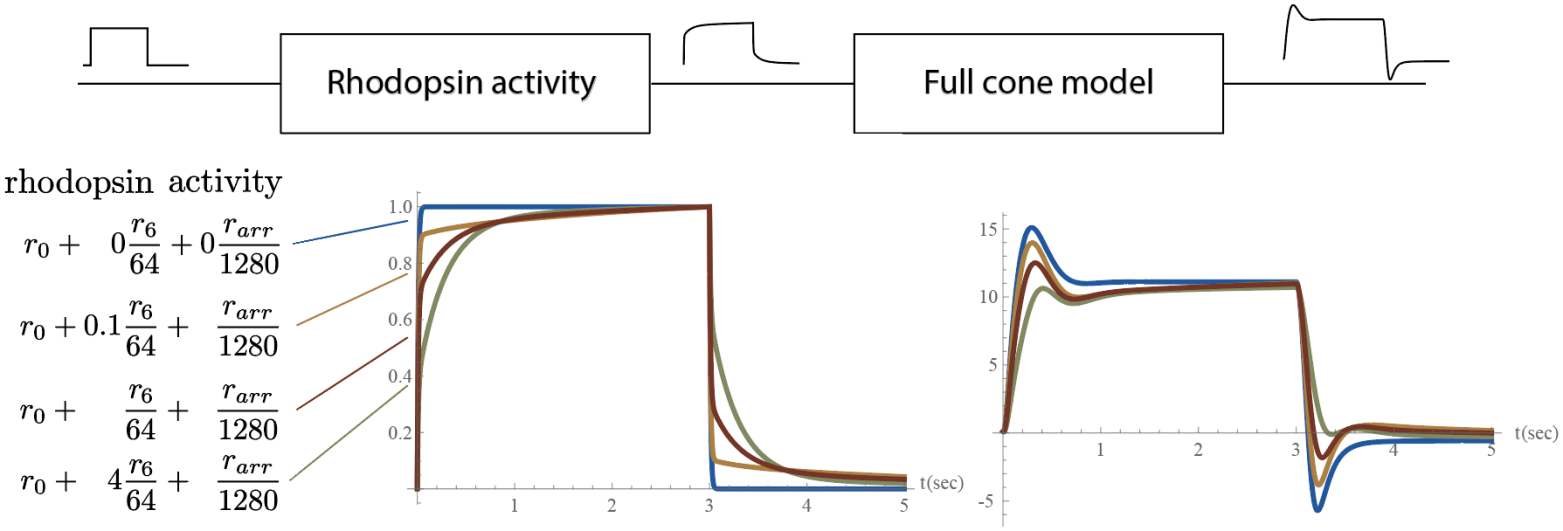
Step responses and responses of the full cone model [15] to them. Blue curves: only *r*_0_. Orange curves: insufficient *r*_6_ activity. Brown curves: approximation to fractional integral. Green curves: excessive *r*_6_ activity.

Fractional components allow the fine-tuning of responses in proportional-integral-derivative (PID)-type controllers [44]. We hypothesise that this rhodopsin signalling behaviour confers additional benefits when combined with the full cone model described in [15]. The full cone model can be considered as a PD-type controller that follows a signal with an overshoot. Fig 6 demonstrates the hypothetical cases where the model parameters are insufficient for approximate fractional integration. Furthermore, the subsequent stages in the retina process the signal and transform the it even further into a derivative of the original signal [45–47]. To demonstrate why this is important, we have plotted three cases in addition to a simple feedback loop: (i) approximation, (ii) when the activity of phosphorylated rhodopsin is too low for approximation, and (iii) where it is too high for an approximation. In this specific case, the inclusion of fractional integrals in the model decreases this overshoot while still allowing a rapid response. However, how this specifically affects the rest of the signalling process remains unknown.

Research on image processing based on visual process (such as this paper or [45, 48–50]) help us understand how human vision works and its differences from the cameras and detectors used in computer vision. As a result, such research will allow us to improve and develop image processing algorithms, and understand the limitations and advantages of not current algorithms and our own vision.
